# Identification of genes associated with shell color in the black-lipped pearl oyster, *Pinctada margaritifera*

**DOI:** 10.1186/s12864-015-1776-x

**Published:** 2015-08-01

**Authors:** Sarah Lemer, Denis Saulnier, Yannick Gueguen, Serge Planes

**Affiliations:** Laboratoire d’Excellence “CORAIL”, USR 3278 CNRS-CRIOBE- EPHE, Perpignan, France, Papetoai, Moorea French Polynesia; Present address: Department of Organismic and Evolutionary Biology, Museum of Comparative Zoology, Harvard University, 26 Oxford Street, Cambridge, MA 02138 USA; Ifremer, UMR 241 EIO, Laboratoire d’Excellence “CORAIL”, BP 7004, 98719 Taravao, Tahiti French Polynesia; Present address: Ifremer, UMR 5244 IHPE, UPVD, CNRS, Université de Montpellier, CC 80, F-34095 Montpellier, France

**Keywords:** Differential expression, Biomineralization, Nacre, Pearl, Pigmentation, Albino

## Abstract

**Background:**

Color polymorphism in the nacre of pteriomorphian bivalves is of great interest for the pearl culture industry. The nacreous layer of the Polynesian black-lipped pearl oyster *Pinctada margaritifera* exhibits a large array of color variation among individuals including reflections of blue, green, yellow and pink in all possible gradients. Although the heritability of nacre color variation patterns has been demonstrated by experimental crossing, little is known about the genes involved in these patterns. In this study, we identify a set of genes differentially expressed among extreme color phenotypes of *P. margaritifera* using a suppressive and subtractive hybridization (SSH) method comparing black phenotypes with full and half albino individuals.

**Results:**

Out of the 358 and 346 expressed sequence tags (ESTs) obtained by conducting two SSH libraries respectively, the expression patterns of 37 genes were tested with a real-time quantitative PCR (RT-*q*PCR) approach by pooling five individuals of each phenotype. The expression of 11 genes was subsequently estimated for each individual in order to detect inter-individual variation. Our results suggest that the color of the nacre is partially under the influence of genes involved in the biomineralization of the calcitic layer. A few genes involved in the formation of the aragonite tablets of the nacre layer and in the biosynthesis chain of melanin also showed differential expression patterns. Finally, high variability in gene expression levels were observed within the black phenotypes.

**Conclusions:**

Our results revealed that three main genetic processes were involved in color polymorphisms: the biomineralization of the nacreous and calcitic layers and the synthesis of pigments such as melanin, suggesting that color polymorphism takes place at different levels in the shell structure. The high variability of gene expression found within black phenotypes suggests that the present work should serve as a basis for future studies exploring more thoroughly the expression patterns of candidate genes within black phenotypes with different dominant iridescent colors.

**Electronic supplementary material:**

The online version of this article (doi:10.1186/s12864-015-1776-x) contains supplementary material, which is available to authorized users.

## Background

Color polymorphisms of mollusk shells have fascinated humans throughout history. Color polymorphism results from a combination of multiple factors starting from inner genetic to mitigating environmental factors related to biochemistry, substrate or nutrition [1, 2]. Experimental crosses have shown that shell color has a genetic basis [3–5]. Most studies have reported a relatively simple genetic basis for color polymorphisms involving one or two loci with dominance [6–8], although more elaborate polymorphisms are likely to arise from complex multigenic systems [9, 10]. Recent studies have shown that marine shellfish tend to display more complex patterns of pigmentation than freshwater shellfish [11]. Examples of complexity are mostly found on shells displaying a nacreous layer (i.e. mother of pearl) such as the gastropods in the family Haliotidae (abalone), the cephalopod family Nautlidae (chambered *Nautilus*), or the bivalves of the families Pteriidae, Mytilidae and Pinnidae. Because of its high industrial value, the composition and formation of the nacreous layer have been studied in multiple taxa at different levels of expertise: proteomics [12–21], genetics [22–26, 21], optics [27, 28], mineralogy [29] and chemistry [30–32]. The nacre is made of regular layers of aragonite tiles and is unique from its iridescence. Iridescence is usually attributed to a diffraction effect caused by the evenly grooved surface microstructure, similar to that of a diffraction grating [33, 34, 27]. The rainbow-like diffraction colors arise from surface repetition of the nacreous layers (growth lines) and are named by gemologists “orient” [29]. However, not all iridescences arise from surface regularities and the pigments present in the binding regions of the aragonite tiles and integrated in the biomineralization process also play a role in the color of the nacreous layer [29]. Therefore the process of biomineralization of the nacreous layer is of great economic interest to the pearl aquaculture industry. As a result, extensive studies have been conducted to identify proteins responsible for the nacre formation by screening proteins contained in the shell and genes specifically expressed in the calcifying tissues i.e. the mantle [35, 36].

**Fig. 1:**
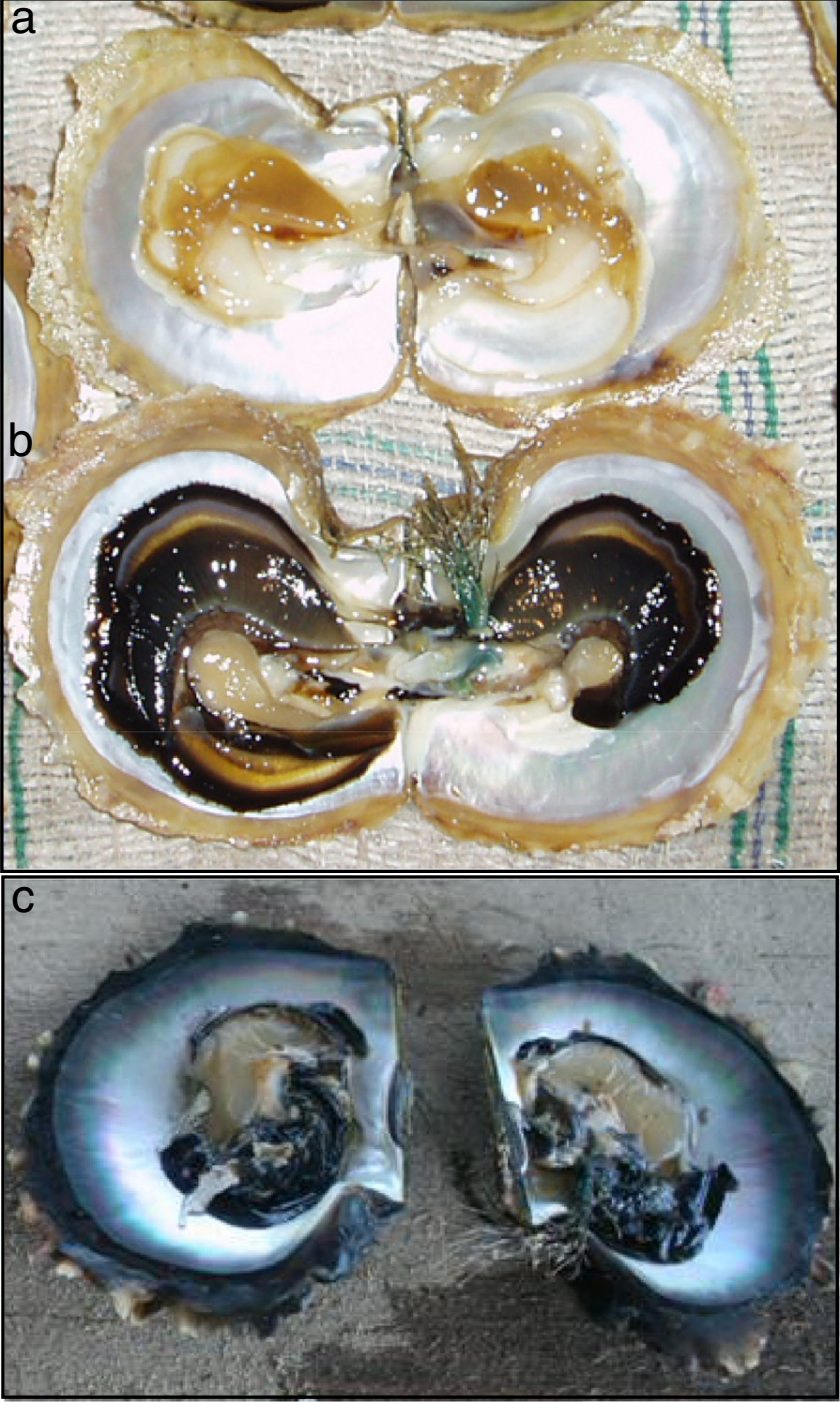
Picture of dissected specimens of *Pinctada margaritifera* of the three studied phenotypes. **a**. Full albino (*FA*; white shell and white mantle), **b**. half albino (*HA*; white shell and black mantle), **c**. black phenotype (*C*; black shell and black mantle)

A wide variety of proteins and genes have been identified and their functions in nacre formation have been partially characterized (reviewed in [35, 37–41]). Our knowledge of the “biomineralization toolkit” involved in the formation of the calcitic and aragonitic layers is expanding and we now know more about how the different shell layers are synthesized and how their production is genetically regulated [37]. Nevertheless the precise molecular mechanism controlling color polymorphism in nacre remains unexplored and is far from being understood. To address this key question we searched for specific genes displaying differential expression patterns among different shell color phenotypes of the Polynesian black-lipped pearl oyster, *Pinctada margaritifera*. The nacreous layer of *P. margaritifera* exhibits a large array of color variation among individuals, although the shell is usually simply referred to as black. The heritability of nacre color variation patterns in *P. margaritifera* have been demonstrated by experimental crossing and by maintaining single colored dominated lineages (CL. Ky personal communication). An interesting feature of *P. margaritifera* is the rare occurrence of albinos, 1 out of 10,000, displaying total or partial absence of coloration. Albinos are characterized by a white shell (periostracum and calcitic layer) and a white mantle (Fig. 1a) in opposition to the common black shells (Fig. 1c). In some rare cases the mantle of albino specimens remains black (S. Planes and S. Lemer, pers. observation; [42]; Fig. 1b); supporting the multiple genetic origins of the absence of color in the shell. In the context of our study, the comparison of albino specimens and black specimens is an ideal method to identify the genetic processes directly linked to shell color polymorphisms.

Albinism is induced by the absence of melanin or by non-functional melanin molecules as a result of one or more mutations in genes involved in the melanin biosynthesis chain [43–49]. Melanin has been identified in many mollusk species, including cephalopods, gastropods, and bivalves [50]. In *P. margaritifera*, melanin is secreted in the epithelium of the median and internal fold of the mantle edge and in the pallial area [51]. The mantle edge is directly responsible for the biomineralization of the shell and more specifically of the nacre layer [21]. As a consequence, it is the chosen mantle part used by professional grafters in pearl culture manufacture to isolate grafts , that will be transplanted together with a marble in the gonads of a recipient oyster. Three proteins closely related to the tyrosinase, known to play a role in melanin biosynthesis, have previously been characterized in the pallial or edge region of the mantle of *Pinctada fucata*: OT47, Pfty1 and Pfty2 [52, 53]. The localization of these proteins suggests that they could be integrated in the shell layers including the nacre layer during the biomineralization process.

In this study, we aimed at identifying genes involved in the origin and potentially the polymorphism of color in the nacreous layer of *P. margaritifera*. We hypothesized that the variety of colors found in the nacre is a combined result of variation in expression of genes involved in the formation of the shell’s microstructure and of genes involved in pigmentation. In order to test this hypothesis, we identified and compared coding genes differentially expressed between normal black specimen of pearl oysters and two types of albinos using a suppressive and subtractive hybridization method, that we then tested and validated with RT-*q*PCR runs. We identified genes involved in the formation of the calcitic layer, the aragonite tablets of the nacre and in the biosynthesis chain of melanin suggesting that color polymorphism takes place at different levels of the shell structure.

## Methods

### Biological material

In order to identify genes involved in the origin and potentially in the variation of shell color, mantle tissue of *Pinctada margaritifera* from 3 phenotypes was obtained from adult specimens of similar size and raised in the same pearl farm in the Gambier archipelago (Mangareva, French Polynesia) in 2011. The three sampled phenotypes were: the normal black phenotype of *P. margaritifera* (black shell and black mantle) referred hereafter as phenotype *C* (Fig. 1b); the full albino phenotype (white shell and white mantle) referred hereafter as phenotype *FA* (Fig. 1a); the half albino phenotype (white shell and black mantle) referred hereafter as phenotype *HA*. We sampled five individuals of each phenotype. The mantle edge of each individual was dissected by a professional grafter following the method used for pearl production, in order to insure the use of the specific part of the mantle producing the calcitic and aragonite layers. Each sample was immediately preserved in RNA*later*^™^ RNA Stabilization Reagent (Qiagen, USA) transported to the laboratory and stored at −80 °C.

### RNA isolation and subtractive cDNA library construction

Total RNA from mantle tissue of individuals of phenotypes *C, HA* and *FA* was isolated using TRIzol reagent (Invitrogen) according to the manufacturer’s instructions. The RNA pellet was washed with 70 % ethanol and air-dried, dissolved in DEPC-treated water and stored at −80 °C. Total RNA was purified using the RNeasy Mini Kit (Qiagen, USA).

The suppressive subtractive hybridization technique (SSH) [54] was used to characterize genes involved in the origin of shell color by comparing expression between the phenotypes *C* and *FA*. For the forward library, cDNA from five phenotype *FA* samples was used as tester and cDNA from five phenotype *C* samples was used as driver, and vice versa for the reverse subtractive library. The construction of the libraries, clone sequencing, vector and adaptor trimming and differential screening using dot blot hybridization were outsourced to Rx. Bioscience Ldt (Maryland, USA). Briefly, both forward and reverse subtracted libraries were produced from 2 ng mRNA. First and second strand cDNA synthesis, *Rsa*I endonuclease enzyme digestion, adapter ligation, hybridization, and PCR amplification were performed as described in the PCR-select cDNA subtraction kit manual (Clontech, Palo Alto, CA, USA) with modifications as described in [55] for the Mirror Orientation Selection protocol. Differentially expressed PCR products were ligated into pJetBlunt cloning vector (Fermentas) and transformed in *Escherichia coli* competent cells. For the differential screening by dot blotting, 1000 clones per library were randomly transferred on two nylon membranes and hybridized with *C* and *FA* cDNA probes, respectively. Nylon membranes were then auto-radiographed and superimposed for identification of differentially expressed clones. Among clones showing the most intense differential signal after hybridization to cDNA probes, 960 were randomly selected for sequencing in each library.

### Sequence analysis

For each library, the 960 clones were sequenced and vector trimmed by RxBioScience Ltd. (Maryland, USA). High-quality expressed sequenced tag (ESTs) (>100 bp) were assembled into clusters or identified as unique sequences and used for database searches with the BlastX and BlastN programs on the NCBI server (http://www.ncbi.nlm.nih.gov/BLAST/) and UniProt. (http://www.uniprot.org/blast/) Search of homology was also conducted in an EST bank of *Pinctada margaritifera* [21]. In addition, functional annotation was performed in Blast2Go (http://www.blast2go.com/) where gene information were obtain by blasting sequences in Gene Ontology (http://www.geneontology.org/). Enriched Molecular function GO terms were then uploaded to REVIGO (Reduce + Visualize Gene Ontology http://revigo.irb.hr/) for visualization. REVIGO summarizes the long list of GO terms by removing redundant terms and grouping related terms based on semantic similarity [56]. The EST sequences used in this study have been submitted to the online database (Accession numbers: JZ845577-JZ845610; JZ845790-JZ845792).

### Differential expression validated by quantitative RT-PCR

Quantitative real-time PCR (RT-*q*PCR) was used to analyze the expression profiles of selected genes. Total RNA was extracted and reverse-transcribed (2 μg) using oligo dT primers and the Superscript II enzyme (Invitrogen).

At first, the cDNA from each of the five individuals for each phenotype (*C, FA* and *HA*) were pooled in equal amounts (25 ng/μL each). Quantitative RT-PCR analyses were run on the three pooled samples for all selected genes. The genes showing the most significant differential expression profile among the three phenotypes were then selected to conduct subsequent RT-*q*PCR analysis on each individual sample separately in order to detect individual variability (Table 1).

**Table 1:** Characteristics of the genes selected for the pooled and individual RT-*q*PCR analyses. The proteins, primers and efficiencies values are indicated. The genes selected for the individual analysis are indicated in bold

Gene code	Protein	Primers	Efficiencies %	Genbank accession #
***KRMP***	Lysine (K)-rich mantle protein	Joubert et al. 2014	95	JZ845792
*SHEM 9B*	Shematrin 9	A_shem9b 5’- F CCCCGTATCCTCCATATCC	101	JZ845577
		A_shem9b 3’- R GCTATTACCGGAGTACCCTACG		
***SHEM 1B***	Shematrin 1	C_Shem1b F 5’- CGCTATCGTTGCTCTCATTG	101.00	JZ845578
		C_Shem1b R 3’- TCCACCTCCTCCTCCTCTTC		
***TYR 2A***	Tyrosinase 2	A_tyr2a F 5’- GCGGCTCTACTGTCAAATGG	112.00	JZ845579
		A_tyr2b R 3’- CTGGACCTTTCAGGGACTGG		
*TYR 2C*	Tyrosinase 2	A tyr2c F 5’- CCCGTGGCCTGGATAGTC	101.00	JZ845580
		A tyr2c R 3’- TTTTCTTCCATCACTGCTACATTG		
*FLAV*	Flavonol cinnamoyl CoA reductase-related	A_flav aF 5’- AGCAGGGTTATCACGTCAGG	96.00	JZ845581
		A_flav bR 3’- ATCCTTTGGTGCCTGTGC		
*METH*	Methionine-rich nacre protein	A_Meth bF 5’- ATGCGGAGGATACTGTGCTT	100.00	JZ845582
		A_Meth bR 3’- CGGGGCTGGATAGACTCATA		
*CLP3*	Chitinase 3 protein	A_clp3b F 5’- TCACAAAATGGATCATAACGTACC	94.00	JZ845583
		A_clp3b R 3’- GGACTGCCTTTGAATGTCG		
***PDZ***	PDZ domain protein	A_PDZa F 5’- TGAGCTTCAGAGAGGTGACG	100.00	JZ845584
		A_PDZa R 3’- AACATTTGGTGAGGGTTTGG		
*MP10*	Mantle protein 10	A_MP10d F 5’- ATTATGGACCGGGCAAGC	100.00	JZ845585
		A_MP10c R 3’- GAGGACAGGAACATCAACAGG		
*EGF*	Epidermal growth factor receptor	A_EGF aF 5’- CATTCCCCATCTTCCTCAAA	108.00	JZ845586
		A-EGF aR 3’- GGACTTCCTGGGATGTTGTC		
*PHOS*	Phosphotyrosine protein phosphatase	A_Phos bF 5’- AATTATGACACAGAGGGATCTAAGG	98.00	JZ845587
		A_Phos aR 3’- CATAGAAACCACCACAACATCG		
*YTH*	YTH domain family protein 2-like	A_YTH aF 5’- AATTGAGACACATTCGGTTGG	93.00	JZ845588
		A_YTH aR 3’- TTACCGCTCTCTGTCCTTGC		
***PIF***	Pif177-like protein	A_pif aF 5’- TTTTGAATTACACGACTGCTTTG	93.00	JZ845589
		A_pif aR 3’- TTCAGTAGAACTAACGCTAAATCCAG		
***CHIT***	Chitin synthase	A_Chit aF 5’- AATCCAATTTCCCGCAGTC	90.00	JZ845590
		A_Chit bR 3’- TTGTTGTAGACATTAGCGACGTATC		
*YBOX*	Y-box binding protein	A_Ybox aF 5’- CGTACATCAAACTGCCATTACC	97.00	JZ845591
		A_Ybox aR 3’- CTACATCAAATTCCACCTTCTCC		
*COLL*	Collagen alpha-1 (XI) chain precursor	A_Coll bF 5’- CGTCCAATCAATCCAGGTG	105.00	JZ845592
		A_Coll aR 3’- TGGGTCCAAAAGGTGAAATG		
*GIGA*	Gigasin	A_Giga aF 5’- TCTCTTCCGGTCAAAAATGC	91.00	JZ845593
		A_Giga aR 3’- TCGCTGTACTATTTCCGTTCG		
***SHEM 4***	Shematrin 4	A_Shem4a F 5’- GCTTCCCATCGGTTTATGG	92.00	JZ845594
		A_Shem4b 3’- R TGCCAACATTTCCGTATCC		
*CAL*	Calreticulin	A_Cal aF 5’- TCACCATCCATTTCATCATCC	102.00	JZ845595
		A_Cal aR 3’- ACCAGAGGACTGGGACAAGC		
*PEROX*	Peroxidase	A_Perox aF 5’- TGCTGGGACTCACTCTATCCA	107.00	JZ845596
		A_Perox bR 3’- TCAAGCCATCAAAGAAACATCTT		
***MP8***	Mantle protein 8	Joubert et al. 2014	101.00	JZ845791
*ASP*	Aspein shell matrix protein	Joubert et al. 2014	99.00	JZ845790
*MAT*	Matrilin Cartilage matrix protein	A_Mat aF 5’- TTGTTCTGGATGGGTCTTCG	109.00	JZ845597
		A_Mat aR 3’- TCAAAGCCCGCACATCAG		
*SHEM 8*	Shematrin 8	C_Shem8 bF 5’- CTCCACCACCAATGACGATT	111.00	JZ845598
		C_Shem8 aR 3’- TTTCGGGGGTGTTAACGTAG		
*FER*	Ferritin	C_Fer cF 5’- GGGCCTGATTGACAGACTTCT	96.00	JZ845599
		C_Fer cR 3’- GAGGGCGCATTGTCCTTC		
*PERL*	Perlin matrix protein	C_Perl bF 5’- TTCCAGATATTACACCCTGTGCT	100.00	JZ845600
		C_Perl aR 3’- CGTTACCGTTTCCACCAAAA		
***PRISM***	Prism uncharacterized shell protein	C_Prism aF 5’- TGGTTCCAAGTGTATTTGTCCA	96.00	JZ845601
		C_Prism aR 3’- TCCATAGACGCACACCTTTG		
*MP88*	molluscan prismatic and nacreous layer 88	C_MP88a F 5’- TCTGTGAAAATTTTGATAAACTGAA	109.00	JZ845602
		C_MP88a R 3’- GAATAAAAGTTTAATGTTCCATTCCT		
*CHK1*	Checkpoint-like protein	C_CHK1a F 5’- CGGGCAGGTACTCATTCC	95.00	JZ845603
		C_CHK1b R 3’- GCTACCACATCCAAGGAAGG		
*LAMIN*	Laminin receptor precursor	C_Lamin bF 5’- GAAGCTGAGAAAGAAGAACAGACC	101.00	JZ845604
		C_Lamin aR 3’- TGTTGTGGTGGCTGTATTGC		
*SEST*	Sestrin 1-like protein	C_Sest aF 5’- TTTTTGCTACCAGGACTTTGC	91.00	JZ845605
		C_Sest aR 3’- GTGTCAACATCTGTCATCTCACC		
*FIBRO*	Fibronectin 3	C_Fibro aF 5’- AGTAGCTGATCGTGCATTCG	97.00	JZ845606
		C_Fibro bR 3’- AACTCACAGGACACCAAAACG		
*SHELL*	Nacre uncharacterize shell protein 6	C_Shell_cF 5’- CGTTCTATCCCTGGTCATCC	101.00	JZ845607
		C_Shell_dR 3’- GGACGTGGATTTTCCTTGG		
***SERP***	Serine protease inhibitor	C_SerP_cF 5’- AGGTGTGTACCATTCTTCTACGG	92.00	JZ845608
		C_SerP_cR 3’- GCAAACATCTCCTCCATCTCC		
***ZINC***	Zinc metalloprotease	C_Zinc bF 5’- CAGAGATGGTTTTGTGTTACTTACG	105.00	JZ845609
		C_Zinc aR 3’- GCTTTGAGGCATTCATGTCC		
*TRANS*	Translocon-associated protein subunit gamma-like	C_Trans aF 5’- TGCCCTAAAGAGGGAAAGC	102.00	JZ845610
		C_Trans aR 3’- CCACAATTAGCAGCACAAGG		

Both pooled and individual RT-*q*PCR analysis were conducted as the following: in each 25 μl reaction, 10 μl cDNA (diluted 1:100 in water) was mixed with 12.5 μl Brillant® II SYBR Green Master Mix (Stratagene) and 0.2 μM of each primer (primer pairs listed in Table 1). This allowed for the consistent use of standardized thermal cycling conditions performed using a Mx3000P Real-Time PCR System (Stratagene): 95 °C for 10 min, followed by 40 cycles of 95 °C for 30 s, 60 °C for 1 min and 72 °C for 1 min which were found to give efficiencies > 90 % (see below). The RT-*q*PCR reactions were run in triplicate for the pooled analysis and in duplicate for the individual analyses. Melting curve profiles (95 to 55 °C decreasing by 1 °C every 30 s) were assayed to ensure that we were looking at a single product. Each run included three positive cDNA controls also used as interplate calibrators, and three non-DNA template controls (water) for each primer pair and four housekeeping genes: RNA and export factor binding protein 1 or REF 1 [57], 18S [58], Elongation factor 1 or EF1 (EF1S: ATGCTGCCATGGTTGATATG/EF1: GTGGCCTCAGCTTTCTCAAC) and Glyceraldehyde-3-Phosphate Dehydrogenase or GAPDH (GAPDH1S: AGGCTTGATGACCACTGTCC / GAPDH1R: AGCCATTCCCGTCAACTTC). EF1 and GADPH were chosen based on their ubiquitous and constitutive expression pattern in *P. margaritifera* tissue.

A 10-fold dilution series was created from a random pool of cDNA from our samples (including *C*, *FA* and *HA* phenotypes), ranging from × 100 dilutions to × 100,000 dilutions. Triplicate RT-*q*PCR reactions were carried out as described above for each gene at each dilution. Mean cycle threshold (C*t*) values for each dilution were plotted against the log10 of the cDNA input for each gene to generate efficiency plots. The reaction efficiency of each gene assay was calculated using the following equation: E = 10(−1/slope) where E is the reaction efficiency and ‘slope’ is the slope of the line generated in the efficiency plots. Only the genes yielding an efficiency comprised between 90 % and 110 % were retained.

### RT-*q*PCR data analysis

The C*t* values of the replicates for each housekeeping gene and each candidate gene of each phenotype (*C, FA* and *HA* pooled or individual samples) were reported. For each candidate gene, the level of transcription was normalized using the following calculation:

$$ \Delta \mathrm{C}t=\mathrm{C}{t}_{\mathrm{Target}}-\mathrm{C}{t}_{\mathrm{M}} $$

where C*t*_Target_ is the mean C*t* of the target gene in one of the tested phenotype (*C, FA* and *HA* pooled or individual samples) and C*t*_M_ is the mean C*t* of the housekeeping genes in that same phenotype.

Relative quantification of gene expressions was estimated for each gene in each phenotype using the ΔΔC*t* method as described in [59]. Relative quantification relates the PCR signal of the target transcript (here *FA* or *HA* phenotypes) in a treatment group to that of another sample (here *C* phenotype). We used the following equation:

$$ \Delta \Delta \mathrm{C}t\kern0.5em =\kern0.5em \Delta \mathrm{C}{t}_{\mathrm{Target}}\kern0.5em -\kern0.5em \Delta \mathrm{C}{t}_{\mathrm{C}} $$

where ΔC*t*_Target_ is the ΔC*t* obtained for a target gene in one of the tested phenotype (*FA* and *HA* pooled or individual samples) and ΔC*t*_C_ is the ΔC*t* obtained for that same gene in the phenotype *C*. In the pooled analysis ΔC*t*_C_ is the ΔC*t* of the pooled *C* individuals whereas in the individual analysis the ΔC*t*_C_ is the mean ΔC*t* obtained for each individual from the *C* phenotype.

Finally the relative expression fold for each gene in each tested condition in relation to the phenotype *C* was estimated using the following equation:

$$ {\mathrm{E}}^{*}{2}^{-\left(\Delta \Delta Ct\right)} $$

where E is the reaction efficiency for each target gene.

For each target gene in each tested phenotype of the pooled analysis, a relative expression fold superior to 2 was considered as significantly differentially expressed [60, 61]. For the individual analysis, significances among mean relative expression folds of each gene were determined by an analysis of variance (ANOVA).

## Results

### Subtractive cDNA library construction and sequencing

Two SSH libraries were produced to identify differentially expressed genes between normally black colored and full albinos specimens of *Pinctada margaritifera*. Nine hundred and sixty (960) clones were sequenced from each library, yielding 677 and 580 high quality cDNA sequences (>100 bp), after removing low-quality regions and screening for vector contamination, from the *C* and *FA* libraries respectively. The ESTs from the *C* library generated 120 contigs and 238 singletons (358 total analyzed sequences after discarding redundant sequences), indicating that the overall redundancy of the library was 18 %. The ESTs from the *FA* library generated 82 contigs and 264 singletons (346 total analyzed sequences after discarding redundant sequences), indicating 54 % redundancy of this library. The average length of the ESTs was 521 bp and 781 bp in the *C* and *FA* libraries, respectively (Table 2).

**Table 2:** General characteristics of the black (*C*) and Full Albino (*FA*) SSH libraries

	*C*	*FA*
Sequenced clones	960	960
Retained sequences	677	580
Analyzed cDNA	358	346
Mean EST size (bp)	521	781
Contigs	120	82
EST in contigs	439	316
Singletons	238	264
Redundancy (%)	18	54

Singletons and consensus clusters were subjected to BLATX searches in UniProt; 58 % of all the blasted sequences from the *C* library and 59 % from the *FA* library obtained a sequence description. Most of the sequences of known gene function presented similarities to predicted proteins from unknown species (records that have no known mapping to the NCBI taxonomy) and *P. margaritifera*.

**Fig. 2:**
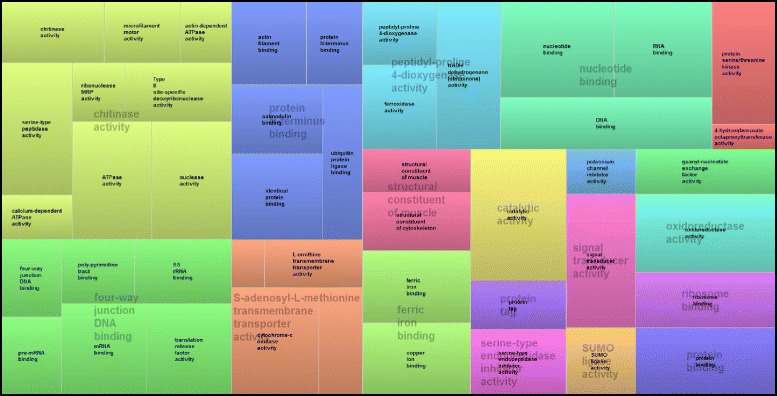
TreeMap visualization of the summary of GO Molecular function found for the black phenotypes, obtained with a Revigo analysis. The size of each boxe is correlated to the frequencies of the occurence of the GO-term. Boxes with the same color are grouped by semantic similarity (SimRel, similarity = 0.7) and correspond to the same upper-hierarchy GO-term which is found in grey in the middle of each box

**Fig. 3:**
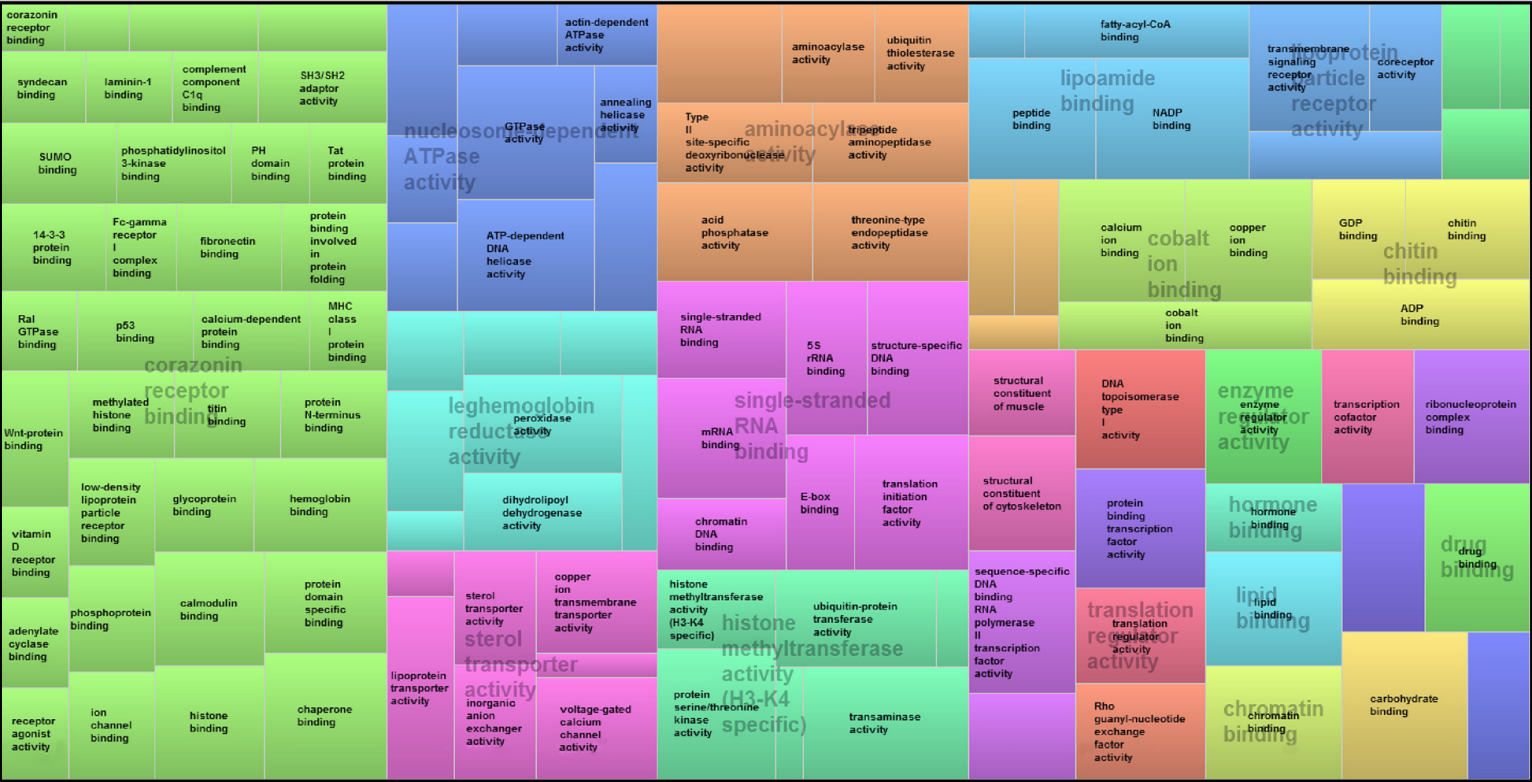
TreeMap visualization of the summary of GO Molecular function found for the full albinos phenotypes obtained with a Revigo analysis. The size of each boxe is correlated to the frequencies of the occurence of the GO-term. Boxes with the same color are grouped by semantic similarity (SimRel, similarity = 0.7) and correspond to the same upper-hierarchy GO-term which is found in grey in the middle of each box

The 358 sequences obtained from the *C* library and the 346 sequences issued from the *FA* library were separately annotated and classified according to the terms of the main Gene Ontology vocabulary for molecular function level 3, using the Blast2GO software. 18.9 % of the ESTs from the *C* library were related to the structural constituent of ribosome category (See Additional file 1). In both libraries, a large number of categories were attributed to binding (57 % and 66 % of the ESTs from the *C* and *FA* libraries respectively, Figs. 2 and 3); which includes protein and ion binding, organic cyclic and heterocyclic compound binding, transcription factor and DNA binding (Figs. 2 and 3). Overall, 23 categories of genes were solely found in the *FA* library, including functions such as transcription factor activity, extracellular matrix binding or metalloenzyme regulator activity (Fig. 3 and Additional file 2).

### Confirmation of differentially expressed genes by RT-*q*PCR

Quantitative real-time PCR experiments were carried out to validate gene differential expressions and to search for differential expression among the black (*C*), full albino (*FA*) and half albino (*HA*) phenotypes. Thirty seven genes (37) associated with aragonite formation, periostracum and calcite biosynthesis, shell protein matrix formation, melanin biosynthesis, ion binding, oxidase activity and transcription factors were selected to perform the expression analyses on the pooled individuals (Table 1).

**Fig. 4:**
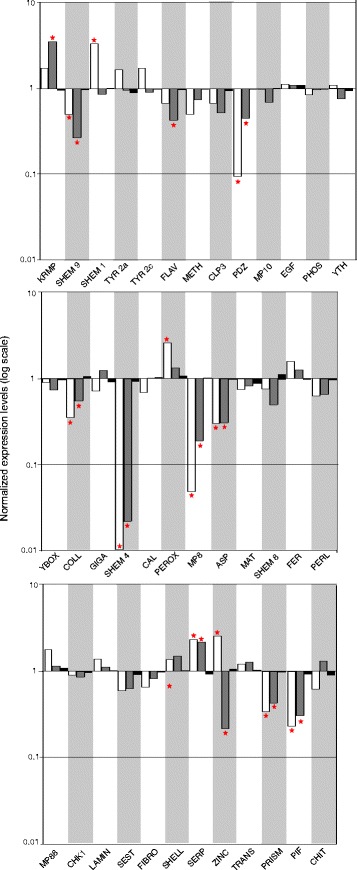
Relative gene expression levels obtained with the pooled analysis. For each tested gene, the white histogram represents the expression in the full albino phenotype (*FA*), the grey histogram in the half albino (*HA*) and the black histogram in the black phenotype (*C*). TYR2a and TYR2c are 2 fragments of the same gene coding for Tyrosinase. Significant differential expressions (>2 folds) in phenotypes (*FA*) and (*HA*) relative to phenotype (*C*) are indicated by a red star

The RT-*q*PCR performed on the pooled individual in each of the three phenotypes revealed that four genes were found to be significantly up-regulated in the *FA* phenotype (*SHEM 1*, *PEROX*, *SERP* and *ZINC*) and two in the *HA* phenotype (*KRMP* and *SERP*) relatively to the *C* phenotype (one being shared with the *FA* phenotype; Fig. 4). The expression of eight other genes was down-regulated in the *FA* and *HA* phenotypes (*SHEM 9*, *PDZ*, *PIF*, *COLL*, *SHEM 4*, *MP8*, *ASP* and *PRISM*). In addition, the expression of 2 more genes were down-regulated in the *HA* phenotype (*FLAV*, *ZINC*) relatively to the *C* phenotype. Within the five up-regulated genes in the *FA* and *HA* phenotypes, two are known to play a role in the shell calcitic layer biosynthesis (*SHEM 1* and *KRMP*) and one is known to be involved in the shell nacreous layer biosynthesis (*SERP*). Of the 10 down-regulated genes found in the *FA* and *HA* phenotypes, four are known to play a role in the shell calcitic layer biosynthesis (*SHEM 9*, *SHEM 4*, *PRISM* and *FLAV*) and one is known to be involved in the shell nacreous layer biosynthesis (*PIF*). The gene *ZINC*, involved in melanin biosynthesis, was oppositely expressed in the two albino condition: up-regulated in the *FA* condition and down-regulated in the *HA* condition (Fig. 4).

**Fig. 5:**
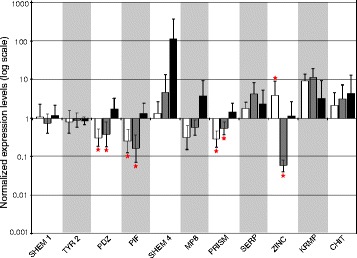
Relative gene expression levels obtained with the individual analysis. For each tested gene, the white histogram represents the expression in the full albino phenotype (*FA*), the grey histogram in the half albino (*HA*) and the black histogram in the black phenotype (*C*). Significant differential expressions in phenotypes (*FA*) and (*HA*) relative to phenotype (*C*) (determined by an ANOVA) are indicated by a red star

Once the previous results were compiled, 9 genes were finally selected for the subsequent individual RT-*q*PCR analysis based on their high differential expression level and their known or predicted role at different levels of the shell formation: *SHEM 1*, *SHEM 4*, *PDZ*, *PIF*, *MP8*, *PRISM*, *SERP*, *ZINC* and *KRMP*. In addition and although they did not show significant differential expression in the pooled analysis, two more genes, *CHIT* and *TYR 2a*, were included in the individual RT-*q*PCR analysis because of their known role in melanin biosynthesis. Some genes showing differential expression patterns in the pooled analysis were however not included in the individual RT-*q*PCR analysis because they belonged to the same sub-family and showed similar expression pattern as another gene selected for this analysis, e.g. *SHEM 9*, *COLL* or *ASP*. The genes *SHEM 9* and *SHEM 4* displayed the same expression pattern and are part of the same shematrin sub-family; as a consequence we choose to analyze only *SHEM 4* and *SHEM 1* which both belong to two different shematrin sub-families. The same reasoning was applied to the gene *COLL* that is supposed to have a similar role as the gene *CHIT*. In addition we tried to select a somewhat equal number of genes involved in the different shell layers (calcitic and aragonite) and in pigmentation, thus excluding the gene *ASP*, involved in the calcitic layer formation and for which we already had selected 4 genes. The RT-*q*PCR performed on each individual of each phenotype separately, revealed the same expression patterns as the pooled analysis for most genes. The gene *SHEM 1* displayed a different expression pattern than the one obtained from the pooled analysis: i.e. absence of differential expression in any tested conditions. The genes *SHEM 4*, *MP8*, *SERP* and *KRMP* failed to display differentially significant expression patterns among phenotypes because of high individual variability in the expression of those genes in the *C* phenotype (which we used as a reference value, Fig. 5). In fact, individual RT-*q*PCR analyses revealed that the relative expression of most targeted genes in the *C* phenotype significantly differed from 1 (except for *SHEM 1*, *TYR* 2a, *PIF* and *ZINC*). This result highlights the high amount of inter-individual gene expression variability in the black specimens (1 being the value corresponding to an absence of variation).

## Discussion

The nacreous layer of the Polynesian black-lipped pearl oyster *Pinctada margaritifera* exhibits a large array of color variation among individuals. In order to study the corresponding molecular mechanisms behind the nacre color variation patterns, a selection of genes differentially expressed between normal black specimens of pearl oyster and two types of albinos were identified using a SSH method and characterized. A subset of these genes encode for proteins involved in shell formation and in the melanin biosynthesis chain.

### Origin of shell color induced by shell matrix protein expressed in the nacre and calcitic layers

The genes *PIF* and *COLL*, directly involved in biomineralization of the nacreous layer, both showed higher expression in the black phenotype compared to both albino phenotypes in all RT-*q*PCR analyses for *PIF* and in the pooled analysis for *COLL. PIF* codes for the gene *Pmarg*-Pif, previously identified by [21]. It is a homolog to Pif-177 found in *Pinctada fucata* [62] and is known to specifically bind to aragonite crystals. Pif-177 is an important component of the nacreous layer and takes part in the initiation of aragonite crystallization as well as subsequent stacking of aragonite tablets in the nacreous layer. Pif-177 also takes part in lamellar sheet formation and therefore it is an essential component of the organic matrix for normal growth of the nacreous layer [62]. Berland [24] showed that the down-regulation of the gene coding for Pif-177 led to a cessation of growth of the nacreous layer. The gene *COLL* (coding for Collagen alpha-1 XI chain precursor) is involved in the growth of collagen II fibrils in humans and seems to be related to chitin in bivalves [63]. Here, we show that both *PIF* and *COLL* are most likely involved in the establishment of color in the shell of *P. margaritifera*. Because the proteins produced by these genes are specifically expressed in the organic matrix and regulate the orientation of the c axis of the aragonite crystals [62, 64], we hypothesize that the color glints observed in the nacre are entailed to the orientation of the aragonite crystals. We however encourage conducting in situ hybridization experiments in the near future to confirm this hypothesis. The weak expression of these genes in the albino phenotypes suggests that the arrangement of albino aragonite crystals differs from those of black shells and contribute to a colorless nacre.

Differential expression patterns were however not detected for all genes involved in nacre formation. The genes coding for the proteins chitin and pearlin showed low expression in the albino phenotypes but this differential expression was not significant (0.6 instead of < 0.5 for under expression). Chitin is a polysaccharide widely distributed in living organisms and is considered to be a fundamental template in biomineralization, as an alternative to collagen [63]. In bivalves, chitin is involved in shell matrix scaffolding. It is the core of the organic framework surrounding the aragonite tablets in the nacreous layer [64]. Pearlin is also known to play a direct role in the formation of the aragonite crystals in *P. maragaritifera* [12, 65–68]. The fact that these two proteins deeply involved in nacre formation are not showing differential expression patterns among phenotypes tends to indicate that the nacre is not the predominant shell layer responsible for the color of the shell of *P. margaritifera*. However, this conclusion has to be regarded with caution as there are many more genes involved in the formation of the nacreous layer that were not explored in the present work and that could display differential expression. In addition, other processes such as post-transcriptional mRNA treatments were not taken into account here. The different expression patterns observed for *COLL* and *CHIT* also suggest that the two amplified fragments code for two distinct proteins playing a different role in the biomineralization process.

On the other hand, our analyses detected differential expression of many genes known to be involved in the formation of the calcitic layer of *P. margaritifera*, suggesting that variation in shell color could primarily take place in the calcitic layer. These genes code for organic matrix proteins produced in the mantle and were found to be highly expressed in the black specimens. Proteins produced in the mantle are secreted into the extrapallial space, where calcium carbonate (CaCO_3_) crystallizes to build unusual microstructures [69]. The mechanism of this process is unknown, but may involve interactions of the matrix proteins and inorganic ions present in the extrapallial space, leading to crystallization of CaCO_3_ and morphogenesis of the species-specific appearance of the shell [69]. Here, we detected high expressions of two genes in the black phenotype, *PRISM* and *MP8*, coding for recently discovered proteins: uncharacterized shell matrix protein 18 and mantle protein 8, both believed to be involved in the formation of the calcitic layer (B. Marin, pers. comm.). Three more genes, homologs of genes found in *P. fucata* and coding for proteins of the shematrin family, *SHEM 4* and *SHEM 9*, and a gene coding for Aspein (*ASP*) also showed high expression patterns in the black phenotypes in the pooled analysis. Shematrin proteins form a family of mollusk proteins exclusively expressed in the mantle, and particularly in the edge region of the mantle [69, 20]. Recent findings suggest that shematrin proteins are synthesized in the mantle edge and secreted into the prismatic layer of the shell, where they are thought to provide a framework for calcification and to be responsible for the toughness of the shell [69]. Aspein is an acidic protein that has a sequence rich in aspartic acid and is expressed at the outer edge of the mantle [70]. It is the shell matrix protein that controls the precipitation of calcite in *P. fucata* and it is the most acidic protein found in molluscan shell matrix protein [71]. The opposite pattern was observed for two genes coding for proteins participating in the formation of the calcitic layer: *KRMP* and *SHEM 1*. The two genes were found to be highly expressed in both albino phenotypes compared to the black phenotype, in the pooled analysis. *SHEM 1* codes for another homolog of the shematrin family: shematrin-1, which was classified as belonging to a different sub-group of shematrins, along with shematrin-2 and −3 in *P. fucata* [69]. These differences imply that they may play a different role in the formation of the calcitic layer and could explain the opposite expression pattern observed for *SHEM 1* compared to *SHEM 4* and *SHEM 9* in *P. margaritifera*. KRMP stands for lysine (K)-rich matrix protein and codes for a matrix protein family participating in the framework formation of the prismatic layer [72].

The high expression of *PRISM*, *MP8*, *SHEM 4*, *SHEM 9*, and *ASP* and the weak expression of *SHEM 1*and *KRMP* in the black phenotypes suggests that the origin of color in the nacre is under the influence of genes directly involved in biomineralization of the calcitic layer. The two opposite expression patterns observed between genes highlights the complexity of this genetic process.

### Role of pigments in the origin of shell color

A gene not directly linked to the calcification process, *ZINC* displayed a significantly higher expression pattern in full albino (*FA*) phenotypes compared to half albino (*HA*) and black phenotypes (*C*). *ZINC* stands for a gene coding for a protein with a metallic zinc ion binding and catalytic domain: Zinc metalloprotease. This gene is a homolog of Tyrosinase related protein 1 [73] which is known to be involved in the melanin biosynthesis pathway: a tyrosinase-like protein detected in the periostracum layer of *P. fucata* by [52] and named OT47. Tyrosinases are considered to be involved in many biological activities of mollusks, including native immune response [74, 75], formation of egg capsules [76–78], byssus [76, 79, 80], shell matrix proteins [76, 81, 82], and periostracum [76, 83]. OT47 was found to be expressed in the middle fold of the mantle edge of *P. fucata* supporting the assumption that it contributes to the periostracum development. The periostracum is an uncalcified layer covering the outer surface of mollusk shell. It is among the strongest mechanically and the most chemically inert structures in the animal kingdom [76]. In addition to the directly protective contribution to the organism, the periostracum is also thought to play a significant role in shell biomineralization [84]. The periostracum encloses the extrapallial space on the ventral side and isolates it from the external environment, enabling the formation of supersaturation conditions, which is a requisite for the formation of calcified layers [85, 86]. Previous studies also showed that it could serve as a substrate for the initial deposition of calcium and even influence the prismatic layer formation [84, 85, 87–89].

Albino phenotypes are supposed to be mainly the result of the absence of melanin biosynthesis or of synthesis of non- or partly functional melanin protein [44, 90]. The high expression of *ZINC* in the full albino phenotype compared to normal black and half albino phenotypes suggests that the full albino phenotype might overcompensate for a nonfunctional melanin protein by over-expressing the gene coding for this protein. Finally, *FLAV* (coding for Flavonol Cinnamoyl COA reductase related) was found to be highly expressed in the black phenotypes, in the pooled analysis. Flavonol Cinnamoyl COA reductase is an enzyme that contributes in the phenylpropanoid biosyntheis in plants and is enhanced for acclimatization processes at different levels of photosynthetic photon flux [91–94]. Mutations in the gene coding for flavonols in angiosperms have shown to induce dramatic color changes in the flower organs [95]. The gene we detected in *P. magaritifera* is related to the gene coding for this enzyme and although no information is available about its role in animals, we can hypothesize that a homologous function to acclimatization to various levels of light can be found in *P. margaritifera* as the over-expression of this gene is correlated with the presence of a black mantle (which translates into melanin pigment biosynthesis in these individuals).

The expression patterns of genes involved both in the biomineralization of the periostracum and the synthesis of melanin suggest once again that the origin and polymorphism of the color in the nacre of *P. margaritifera* takes place in all the layers of the shell and in different biological processes.

### Differential expression of genes not directly involved in biomineralization processes

Two genes coding for proteins involved in metabolic processes showed significant differential expression among phenotypes: *SERP* and *PDZ*. The gene *SERP* coding for Serine protease inhibitor or serpins (which is part of a large family of enzymes that inhibit the activity of proteases) showed high expression levels in both albino conditions, in the pooled analysis. A limited number of serpins proteins has been reported in bivalves or mollusks and so far they are thought to be involved in an immune defensive role against invasive pathogens [94, 96]. The gene coding for a PDZ domain protein (*PDZ*) showed significantly higher expression levels in black phenotypes than in albino phenotypes, both in the pooled and individual analyses. PDZ domains are one of the most frequently encountered domains, consisting of approximately 90 amino acid residues and identified as a region of sequence homology among a diverse list of signaling proteins [97, 98]. The reason why these genes exhibit differential expression patterns across phenotypes remains unclear and will need further explorations.

### Inter-individual variability in gene expression within black phenotypes

One major outcome of the individual analysis performed on 11 genes is the existence of high gene expression variability within the black phenotypes for most of the genes tested and mostly for *SHEM 4*, *MP8*, *KRMP*, *CHIT* and *SERP*, characterized by high standard deviation values and gene expression levels different than 1 (1 being the value corresponding to an absence of variation ; Fig. 5). It is well known that what is commonly named as the black-lipped pearl oyster is in fact a pool of a wide range of shell colors varying from several shades of yellows, reds, greens and blues. The black specimens used in this study as a control phenotype for the individual analysis, were selected randomly. The inter-individual gene expression variation detected within those samples highlights the diversity of colors found in our pool of black phenotypes. This result supports the hypothesis that *SHEM 4*, *MP8*, *KRMP*, *CHIT* and *SERP* are very likely to be involved not only in the origin of shell color but also in the variability of the shell color among "black" specimens. The presence of these wide variations in expression patterns in the black phenotypes, used as controls in this analysis, could also be responsible for preventing the detection of significant differential expression patterns in the individual analyses of albinos specimens when those patterns were significant in the pooled analysis.

## Conclusions

The goal of this study was to identify a subset of genes involved in the color of the nacreous layer of the pearl oyster *Pinctada margaritifera,* and assess their expression discrepancies*.* The role of the proteins encoded by the genes showing differential expression levels among the different tested phenotypes revealed that three main genetic processes were involved in color polymorphisms: the biomineralization of the nacreous and calcitic layers and the synthesis of pigments such as melanin in the periostracum. However, our results suggest that the color observed in the nacre is mainly under the influence of genes involved in the biomineralization of the calcitic layer. A few genes involved in the formation of the aragonite tablets of the nacre and in the biosynthesis chain of melanin also showed differential expression patterns, suggesting that color polymorphism takes place at different levels in the shell structure. Color in various organisms like birds or flowers is known to be under the influence of a combination of genes coding for multiple pigments (melanin and carotenoid being the most commons in birds and anthocyanin in angiosperms) and surface structure [99, 95]. Different gene expression arrangements seem to lead to the extraordinary variety of colors and iridescence observed in bird feathers and flower organs [99, 95]. Similarly, it appears that the genes involved in the origin and variation of color in the Polynesian black-lipped pearl oyster span a large range of functions from shell structure to pigment synthesis. Future studies, using RNA-Seq technology and in situ hybridization analyses of the genes identified in the present work will help define more precisely the role of the different shell layers in the origin of the color of the nacre. The high variability of the gene expression found within black phenotypes furthermore suggests that the present work should serve as a basis for future studies exploring more thoroughly the expression patterns of candidate genes within black phenotypes with different dominant iridescent colors.

### Availability of supporting data

The datasets supporting the results of this article are included within the article and its additional files.

## Additional files

Additional file 1:
**Pie chart visualization of the summary of GO Molecular function level 3 found for the black phenotypes, obtained with the Blast2GO software.** (PDF 4 kb) Additional file 2:
**Pie chart visualization of the summary of GO Molecular function level 3 found for the full albinos phenotypes, obtained with the Blast2GO software.** (PDF 7 kb)

## Abbreviations

ANOVA: Analysis of variance
C: Colored phenotype
CaCO_3_: Calcium carbonate
C*t*: Cycle threshold
E: Efficiency
EST: Expressed sequence tag
FA: Full albino phenotype
HA: Half albino phenotype
SSH: Suppressive and subtractive hybridization
