# Supplementary figures and images for: Identification of genes associated with shell color in the black-lipped pearl oyster, Pinctada margaritifera
Source: BMC Genomics. 2015 Aug 1;16(1):568. doi: 10.1186/s12864-015-1776-x (PMC4521380; doi:10.1186/s12864-015-1776-x)

# molecular\_function Level 3

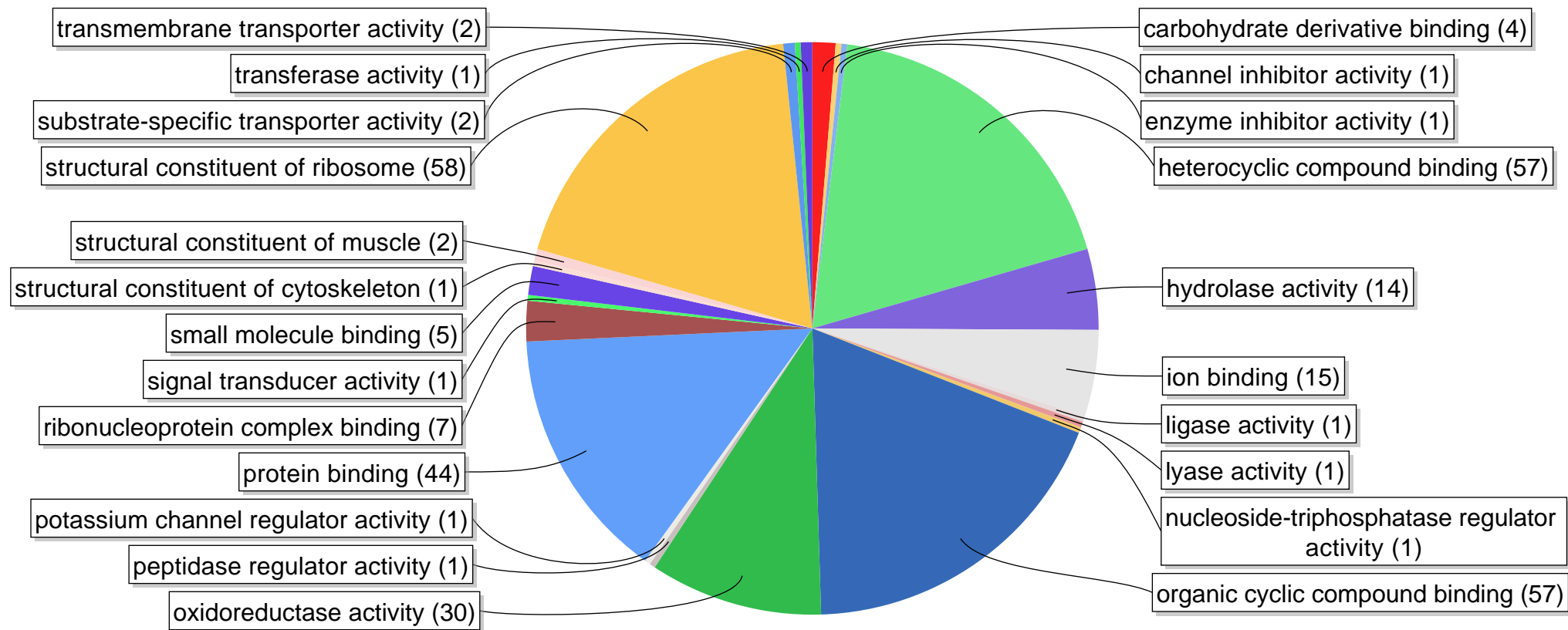

Supplement: Additional file 1: — Pie chart visualization of the summary of GO Molecular function level 3 found for the black phenotypes, obtained with the Blast2GO software. (PDF 4 kb) [file 12864_2015_1776_MOESM1_ESM.pdf]

# molecular\_function Level 3

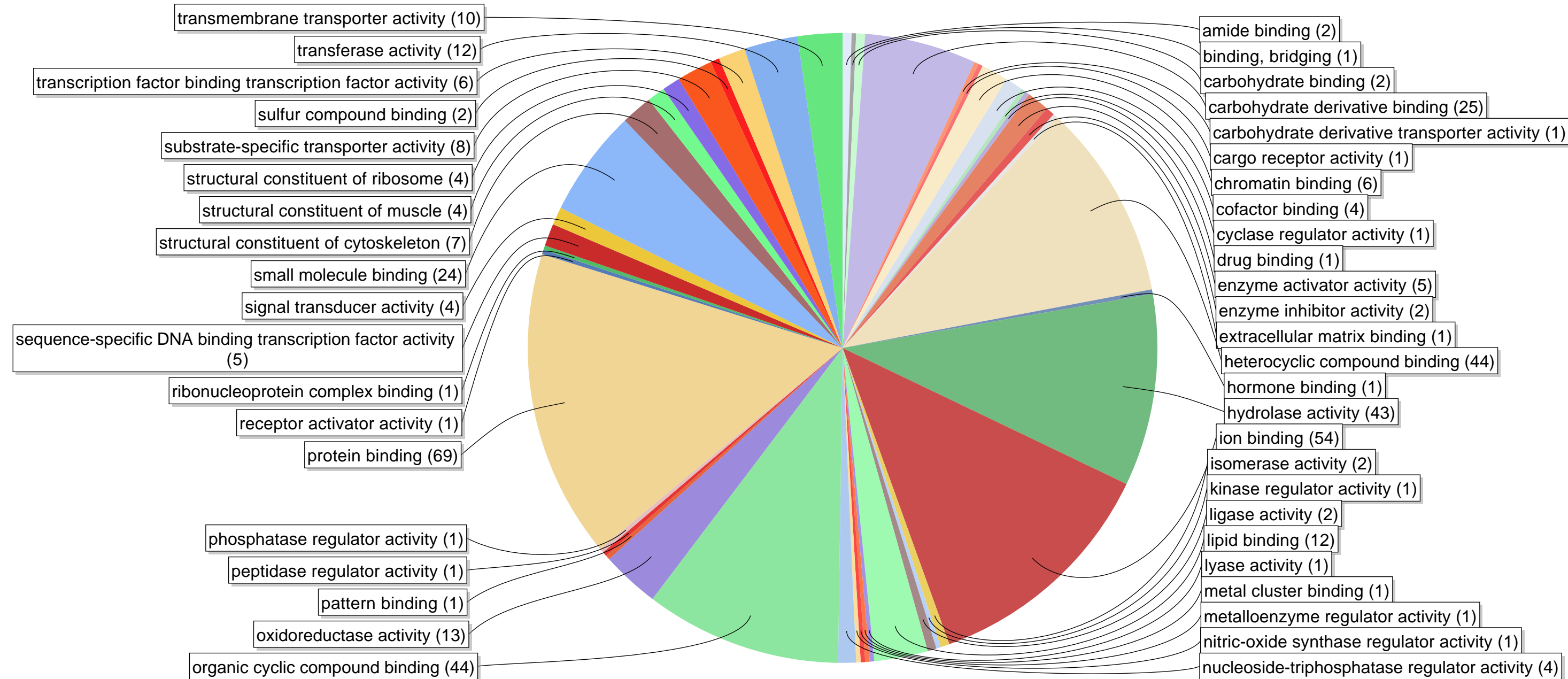

Supplement: Additional file 2: — Pie chart visualization of the summary of GO Molecular function level 3 found for the full albinos phenotypes, obtained with the Blast2GO software. (PDF 7 kb) [file 12864_2015_1776_MOESM2_ESM.pdf]
